# The development of a Biobank of cancer tissue samples from World Trade Center responders

**DOI:** 10.1186/s12967-018-1661-x

**Published:** 2018-10-11

**Authors:** Wil Lieberman-Cribbin, Stephanie Tuminello, Christina Gillezeau, Maaike van Gerwen, Rachel Brody, Michael Donovan, Emanuela Taioli

**Affiliations:** 1 Department of Population Health Science and Policy and Institute for Translational Epidemiology, Icahn School of Medicine at Mount Sinai, One Gustave L. Levy Place, Box 1133, New York, NY 10029 USA; 2 Department of Pathology, Icahn School of Medicine at Mount Sinai, New York, NY USA

**Keywords:** Cancer incidence, Biorepository, September 11th, WTC Health Program

## Abstract

**Background:**

World Trade Center (WTC) responders were exposed to mixture of dust, smoke, chemicals and carcinogens. Studies of cancer incidence in this population have reported elevated risks of cancer compared to the general population. There is a need to supplement current epidemiologic cancer follow-up with a cancer tissue bank in order to better elucidate a possible connection between each cancer and past WTC exposure. This work describes the implementation of a tissue bank system for the WTC newly diagnosed cancers, focused on advancing the understanding of the biology of these tumors. This will ultimately impact the modalities of treatment, and the probability of success and survival of these patients.

**Methods:**

WTC Responders who participated (as employees or volunteers) in the rescue, recovery and cleanup efforts at the WTC sites have been enrolled at Mount Sinai in the World Trade Center Health Program. Responders with cancer identified and validated through linkages with New York, New Jersey, Pennsylvania, and Connecticut cancer registries were eligible to participate in this biobank. Potential participants were contacted through letters, phone calls, and emails to explain the research study, consent process, and to obtain the location where their cancer procedure was performed. Pathology departments were contacted to identify and request tissue samples.

**Results:**

All the 866 solid cancer cases confirmed by the Data Center at Mount Sinai have been contacted and consent was requested for retrieval and storage of the tissue samples from their cancer. Hospitals and doctors’ offices were then contacted to locate and identify the correct tissue block for each patient. The majority of these cases consist of archival paraffin blocks from surgical patients treated from 2002 to 2015. At the time of manuscript writing, this resulted in 280 cancer samples stored in the biobank.

**Conclusions:**

A biobank of cancer tissue from WTC responders has been compiled with 280 specimens in storage to date. This tissue bank represents an important resource for the scientific community allowing for high impact studies on environmental exposures and cancer etiology, cancer outcome, and gene-environment interaction in the unique population of WTC responders.

## Background

Responders to the World Trade Center (WTC) disaster were exposed to soot, benzene and other volatile organic compounds from jet fuels, as well as WTC dust and smoke, which contained asbestos, silica, cement dust, glass fibers, heavy metals, polycyclic aromatic hydrocarbons, polychlorinated biphenyls, and polychlorinated dibenzofurans and dioxins from the burning and collapse of the planes and the towers [1–3]. Immediately following the 9/11 terrorist attack, concerns were raised about the potential cancer risk among the WTC responders, due to their exposures to a complex mix of toxic chemicals that included multiple known and suspected human carcinogens [4–10]. These carcinogens were contained in a complex mixture that changed in concentration over time, and are likely to induce both early (DNA damage, mutation, reduced DNA repair), as well as late events (cell proliferation, chronic inflammation). They may also act on cancer risk and progression through other mechanisms such as reduced immunological competence and epigenetic alterations of gene regulation [11–14]. Furthermore, there may be a long-term increased cancer risk among WTC responders because of changes in cancer-associated behaviors (e.g., overweight/obesity) [15–18] as a consequence of their stressful experience during the disaster [19].

Few studies have investigated cancer incidence in WTC responders. A 2009 study of multiple myeloma cases in WTC responders reported a higher than expected number of cases in responders aged < 45 years old [20]. A 2011 study investigated cancer among 9853 firefighters enrolled in the Fire Deparment of the City of New York (FDNY) WTC Health Program in the first 7 years following 9/11 [21], and reported a higher standardized incidence ratio (SIR) of cancer in exposed compared to non-exposed firefighters [21].

A follow-up study of 20,984 WTC responders linked their data to the tumor registries of New York, New Jersey, Connecticut, and Pennsylvania and identified 575 cancer cases diagnosed from 9/12/2001 to 12/31/2008, 302 of which were diagnosed 6 or more months after the attack [22]. Standardized incidence ratios to compare cancer by site in responders with that predicted for the general population adjusted for age, sex, and ethnicity/race were elevated for all cancer sites combined (SIR 1.15, 95% confidence interval [CI] 1.06–1.25), and for thyroid cancer, prostate cancer, hematopoietic and lymphoid cancers and soft tissue cancers [22].

An updated study of cancer incidence through 2011 among WTC Health Registry enrollees reported increased all-cancer incidence in both rescue and recovery workers (RRW) (SIR 1.11, 95% CI 1.03–1.20) and non-rescue and recovery workers (SIR: 1.08, 95% CI 1.02–1.15) [23]. When compared to the New York State (NYS) population as a reference, RRW have increased cancer incidence for all-sites (SIR 1.11, 95% CI 1.03–1.20), as well as prostate, thyroid and melanoma [23]. The study cautioned on the lack of biological evidence connecting the results to WTC exposure and emphasized the need for follow-up studies [23]. A separate study of head and neck cancer patients reporting WTC exposure at Memorial Sloan Kettering (2002–2017) identified 87 cases over this time period and reported the annual number and proportion of WTC-exposed head and neck cancer patients have steadily increased since 2002 [24].

Taken together, the results of these studies have prompted the CDC to add several cancers to the list of WTC-related health conditions [25] and added evidence to the importance of ongoing studies of the responder population.

Due to the reported increased cancer incidence, there is a clear need to supplement current epidemiologic cancer follow-up with a cancer tissue bank in order to better elucidate a possible connection between each cancer and past WTC exposure. Biospecimen resources and their clinical annotations are among some of the most powerful resources fueling translational research. This work describes the implementation of a tissue bank system for the WTC newly diagnosed cancers, focused on advancing the understanding of the biology of these tumors. This will ultimately impact the modalities of treatment, and the probability of success and survival of these patients.

## Methods

### Definition of the cohort

WTC responders who participated (as employees or volunteers) in the rescue, recovery and cleanup efforts at the WTC sites have been enrolled at Mount Sinai in the World Trade Center Health Program (WTCHP), which is funded under the James Zadroga 9/11 Health and Compensation Act of 2010, on the basis of eligibility criteria including type of duties, site location and dates and hours worked [22]. The full eligibility criteria have been described previously in the literature [26–28]. The medical protocol for the monitoring program includes self-administered physical and mental health questionnaires, as well as a physical examination, laboratory tests, spirometry and a chest radiograph. Participants undergo visits every 12–18 months at WTCHP clinical centers for monitoring [22]. Over 27,000 responders have had at least one monitoring visit in the WTCHP and have consented to aggregation of their data. A total of 20,984 responders have consented to have their records used for medical research. Most of the participants are males (85%), whites (59%), with a range of WTC exposure, but roughly one-third experienced significant exposure to dust [22].

### Identification of cancer cases

The WTC Data Center identifies cancer cases through periodic linkages with the cancer registries of New York, New Jersey, Pennsylvania and Connecticut, which accounts for 98% of the responder residences at time of enrollment in the WTCHP [22]. To complete the linkage, the last name, first name, sex, race/ethnicity, complete date of birth, address at registration, and Social Security number when available (37%) of consented responders enrolled in the WTCHP from 16 July 2002 to 31 December 2008 (*n* = 20,984) were provided to each cancer registry [22]. The full matching methodology has been described in detail elsewhere [22], but only cancer cases validated by one of these four state cancer registries were eligible to participate in this biobank.

### Patients’ recruitment and consent

**Fig. 1:**
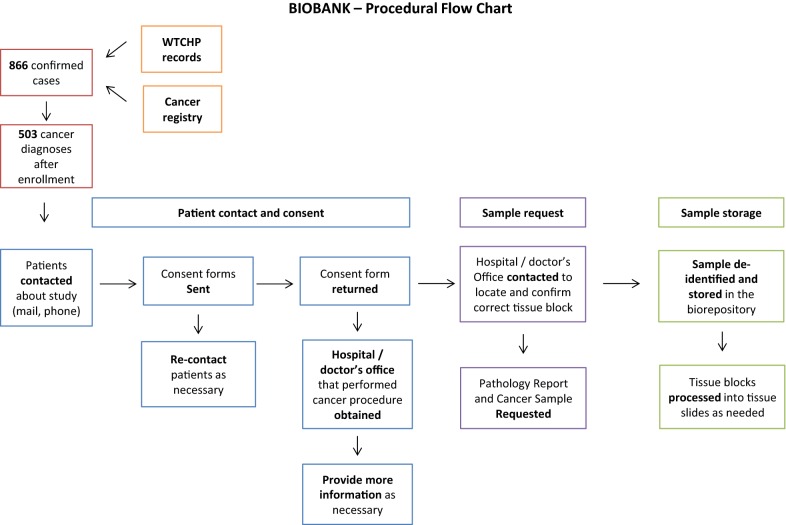
Flow-chart of tissue identification, preparation and storage

Initial letters were sent to potential participants explaining the study and seeking approval to discuss the consent process, with information provided for participants to contact the research team with questions (Fig. 1). After the consent process was discussed, a consent form was sent to obtain written permission to retrieve patient tissue blocks at the hospital where their cancer was diagnosed and staged. As part of the consenting process participants were asked which hospital or doctor’s office performed the procedure (either biopsy or surgery). The consent document also queried about storage of their tissue sample for research purposes. If a response to the initial letter was not received within weeks, phone calls were made and additional letters were mailed to explain the consent process and proceed accordingly. Participants were also contacted through email. Deceased members of the cohort were identified through letters and phone calls. In these cases, family member/next of kin was informed about the study and the need for tissue block retrieval. We currently have an Institutional Review Board (IRB) modification pending that will allow us to ask the permission of the next of kin of those who are deceased for sample retrieval. All participants were mailed a signed photocopy of the written consent they provided. All progress, including dates of contact and the content of interactions was logged in a de-identified and password protected file.

### Tissue procurement and storage

For cases that were not performed at Mount Sinai, relationships were established with the pathology department at the facility where the procedure occurred. Prior to requesting the sample, the pathology department was contacted to identify and confirm the existence of the correct tissue sample of interest. Afterwards, an official sample request was provided to the hospital/doctor’s office together with a shipping label and a request for the patient’s pathology report. The request included instructions about confirming the diagnosis and identifying the appropriate area of malignancy for sampling. If weeks passed without receiving a sample, hospitals and doctors’ offices were re-contacted to sort out any obstacles.

Creating a biobank is a challenge as it is not the policy of many institutions to release their formalin-fixed paraffin-embedded (FFPE) blocks for risk of contamination or the loss of the block. Instead FFPE blocks are sectioned into thin slices using a microtome and placed on glass slides. These slides are sent when samples are requested [29, 30]. Although the specifics of requested slides can vary, charged slides were preferred as their chemical coating more strongly adheres to the sample, which is important for biological applications such as immunohistochemistry (IHC). Requesting charged slides also provided the most flexibility for future assays and research projects. Thus the tissue slide specifications that were requested from each institution were of 4 µm thickness, unstained and on charged slides. When the volume of tumor tissue allowed, a 4 µm tissue curl was also requested. Additionally, for each sample one hematoxylin and eosin (H&E) stained slide was requested; in the cases where an H&E slide was not provided, one of the unstained slides was stained in-house.

Material received from the various institutions was de-identified and a random number was assigned to each tissue block or set of slides received. If necessary, tissue blocks were processed into tissue slides for storage and then returned at the request of the providing hospital or doctor’s office.

Slides were stored in enclosed slide cases in a secure, dry and cool place away from dust and any direct light. It is worth noting that tissue microarrays (TMA) were considered for storage in place of slides but were ultimately decided against. Like tissue slides, TMAs can be constructed from FFPE material and are produced when multiple small tissue “cores” are extracted from different tissue blocks and inserted into one single TMA chip [31]. This cuts down on costs, resources and amount of tissue being used [32]. While this is advantageous for many different types of projects, having hundreds of samples on one TMA block would not have been ideal for this biobank project. While TMA blocks are usually organized according to sample type to reduce the risk of contamination, WTC cancer samples come from a diverse range of cancers. Moreover, when requester research institutions require samples from the biobank they need only specific samples applicable to their respective projects (i.e. only lung cancer samples), and sending the entire TMA to that institution would be a waste of tissue material. Additionally, since institutions rarely send tumor blocks, most samples would still have been cut into slides before being sent to Mount Sinai; creating TMAs from these slides would’ve increased the chance for tissue loss and contamination. TMAs are at similar risk as tissue slides for antigen degradation [32].

## Results

### Inventory

**Fig. 2:**
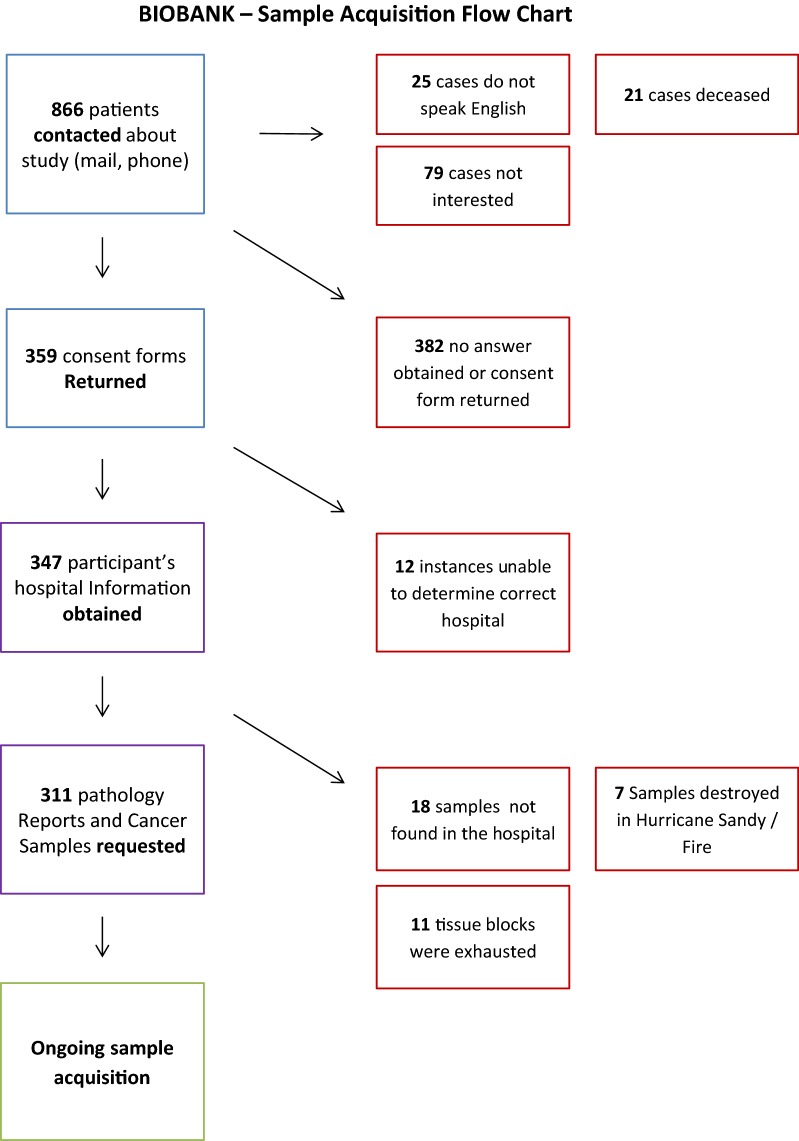
Flow-chart of tissue acquisition

All the 866 solid cancer cases confirmed by the Data Center at Mount Sinai have been contacted and consent was requested for retrieval and storage of the tissue samples from their cancer (Fig. 2). Hospitals and doctors’ offices were then contacted to locate and identify the correct tissue block for each patient. The majority of these cases consist of archival paraffin blocks from surgical patients treated from 2002 to 2015. Samples were collected from 61 institutions. At the time of manuscript writing, this resulted in 280 cancer samples stored in the biobank (Table 1). Forty-five patients had records for two primary cancers, two patients had records of three primary cancers and one patient had records of four.

**Table 1:** Frequency of cancer types included in the biobank

Cancer type	N (%)
Breast	16 (5.7)
Colo-rectum^a^	28 (10.0)
Kidney and renal pelvis	25 (8.9)
Melanoma of the skin	27 (9.7)
Lung and bronchus	24 (8.6)
Thyroid	30 (10.7)
Prostate	62 (22.1)
Head and neck^b^	21 (7.5)
Urinary bladder^c^	17 (6.1)
Other^d^	30 (10.7)
Total	280 (100.0)

^a^Includes: ascending colon, anus, appendix, cecum, hepatic flexure, large intestine, rectosigmoid junction, tubulovillous adenoma, rectum, sigmoid colon, transverse colon

^b^Includes: larynx, lip, nasopharynx, other oral cavity and pharynx, parotid gland, salivary gland, tongue, tonsil

^c^Includes: ureter

^d^Includes: bones and joints, brain, cervix uteri, corpus uteri, esophagus, liver, miscellaneous, thymus, pancreas, small intestine, soft tissue including heart, stomach, testis

### Establishing the tissue bank as a resource for the science community

This biobank serves as a resource available to scientists for etiologic and outcome studies, thus overcoming the historical limitations of other research tissue banks. Access to de-identified clinical data is often complex due to multiple factors including: difficulty of obtaining appropriate control tissues, misconceptions of regulatory and proprietary rules governing tissue banking; specifically IRB, consent, honest broker, de-identification and ownership issues, scarcity of tissues due to lack of cooperation from surgery, pathology, oncology and clinical/translational/basic science researchers who may compete for the same resources.

One of the most important aspects of this project is the availability of the tissue samples to the scientific community, with the purpose of allowing high impact studies on environmental exposures and cancer etiology, cancer outcome, and gene-environment interaction in the unique population of WTC responders. We have established a process for qualified applicants to request available samples for use in research projects, and a mechanism for tissue utilization by creating two structures, the research evaluation panel (REP) and the Coordinating Committee (CC).

### Research evaluation panel to guide tissue utilization

The REP consists of the Principle Investigator of the tissue bank and three other members of the cancer research community and is responsible for determining the importance of the proposed studies, the areas of weakness that require improvement, and for developing recommendations to the CC. The REP review is scientifically rigorous, and a brief written review is requested for internal documentation and to guide discussions with the investigators. The REP convenes regularly by telephone to discuss and approve projects.

### Roles of the Utilization Committee

The Utilization Committee (UC) negotiates the balance between the scientific merit of a proposal as assessed by the REP and sample availability and sustainability for future studies. The UC meets in coordination with the REP and communicates heavily with REP members in order to discuss disagreements and differences in the priorities for sample allocation.

### Roles of the Coordinating Committee

The biobank has a governing CC that includes a member from the Centers for Disease Control and Prevention/National Institute for Occupational Safety and Health (CDC/NIOSH) funding agency, the latter of which acts as a resource for information about CDC activities and advises on the acceptability of the CC’s policies to the CDC. The CDC member also has the unique capability of informing other scientists about the availability of the tissue bank. The CC oversees and guides the tissue bank development and ongoing activities, acts as the governing body of the developing operating policies, and has prepared a Manual of Operations for establishing uniform procedures to accession, process, and distribute the tissue samples. The CC determines priorities for application's final approval based on the recommendation of the REP; it also publicizes the availability of this resource to prospective users.

### Agreement and utilization

After final approval is granted, the research institution requiring the samples must provide IRB approval for the project, and a data-use-agreement between the institution and the biobank must be created. This ensures that tissue samples are only being used for their specific, and approved, purpose. Proposals will only receive de-identified tissue and data, ensuring there is no means to link tissue samples to participants. If two quality research proposals are received for similar projects, we will look for commonality to form collaborations between the two projects, especially if the type and amount of cancer tissue required is limited. Possible limitations of the process include the lack of availability of sufficient amount or type of tissue for the proposed research question, and the possible insufficient number of cases for a certain cancer type.

### Dissemination

Outreach and advertising of the tissue bank is accomplished through partnership with the WTCHP, CDC, the scientific community and the relevant stakeholders and patient advocacy communities. This approach recognizes that the creation of an effective, sustainable resource is dependent on the participation, trust, and involvement of all these key groups. The existence of the tissue bank, as well as any results generated by using the tissue samples is communicated to WTC members, as well as participants in other WTC surveillance programs.

The tissue bank is also presented to the wider scientific community through posters at scientific meetings and articles for publication in the medical literature. De-identified raw data generated within this project are made rapidly available to other WTC researchers and the wider medical and preventive community. In an effort to reach out to the community, we have established a secure cloud-based website within the Mount Sinai server (http://icahn.mssm.edu/research/epidemiology/capabilities/biorepository-wtc) that acts as an information portal modeled on previously developed websites by our group. The website includes general information about the type of specimens available, procedures and requirements for obtaining tissue, as well as the electronic forms necessary for making tissue requests and inquiries. The website also includes a password-protected file transfer site, where letters of intent, documentation, publications and other reports (such as status of applications, etc.) are posted for review by the REP, UC, and CC.

All scientific abstracts, presentations, and publications resulting from use of the tissue bank, and patents or products resulting from use of the tissue bank are recorded in the website. The number of grants submitted and the number of grants funded are also being tracked as a measure of success.

In addition, we have dedicated a full page of the Institute for Translational Epidemiology (ITE) printed brochure to the World Trade Center Biobank. The brochure is currently distributed to major scientific meetings focusing on cancer and epidemiology, and has served as a way to inform the scientific community about the tissue bank.

### Evaluation of the relevance and effectiveness of the tissue bank

Specialized software is used to provide access-log analysis for the website. This software analyzes the log files created by the web server and provides invaluable information on how users access the website, including statistical information as well as color graphs that show trends and usage. The software periodically analyzes the data and generates reports on samples requests, usage, scientific publications deriving from the tissue bank, number of grants submitted and funded, among others.

### Problems encountered and addressed

Initially, there was difficulty in contacting participants by phone because of disconnected numbers, incorrect numbers, and participants not answering their phones or responding to any messages. In some instances, participants had moved or updated their address since their enrollment in the cohort, which prevented the delivery of consent forms. This resulted in a limited number of consent forms returned. However participants were repeatedly contacted multiple times over multiple days at different portions of the day. Home, mobile, and work numbers were used to establish contact together with sending emails to those members of the cohort who had provided their email address. Participants were also contacted by phone in conjunction when consent forms would arrive at their address. This ensured that cases were reminded of the incoming documents and could ask any questions before the forms arrived. Potential participants were also contacted to notify them of an incoming study being mailed to their address.

For procedures not performed at Mount Sinai, participants had to be contacted to receive the information at which hospital/office they had their cancer procedure performed. This provided another hurdle in acquiring samples, but an emphasis was made on assessing hospital information whenever contact was made with participants. Requesting samples from outside institutions required locating and establishing contacts with the appropriate member of the pathology department. This entailed completing different procedures for different institutions, which took time to grow accustomed to. In instances where institutions provided tumor blocks instead of slides, blocks were processed at Mount Sinai and returned to the institution as soon as possible.

### Future directions

The biobank is in the process of updating the current samples with the cancers that were diagnosed after 2014. To increase participation, employees that speak languages other than English will be recruited for the project. In the future we will continue to address patient’s concerns and fears of participating in the biobank to increase recruitment. We are also working on linking the tissue bank with the main WTCHP data set containing clinical, epidemiological and exposure information, both at the time of inclusion in the WTCHP and during the regularly performed follow up, and to the blood sample collected at baseline.

The biobank has also started to standardize and manage a central repository of tissue samples from various organs from rodents exposed to WTC dust; including 376 rats exposed to WTC dusts via intratracheal inhalation [33–36] by NYU. Blood serum and plasma, bone marrow, aortic arch, heart, lung, kidney, liver, spleen, tibialis anterior muscle, and prostate tissue of rodents are stored at − 20 °C, − 80 °C, or in 10% formalin according to standard practice. Relevant information from pathology reports is also centrally stored, along with details of the exposure amount and duration, and of the experimental design. The biobank will facilitate translational studies that will give a comprehensive view of the effect of WTC exposure on cancer etiology, occurrence and aggressiveness. All studies using the WTC tissue bank would benefit from confirming their findings in corresponding tissues from organs of animals experimentally exposed to WTC dust.

**Fig. 3:**
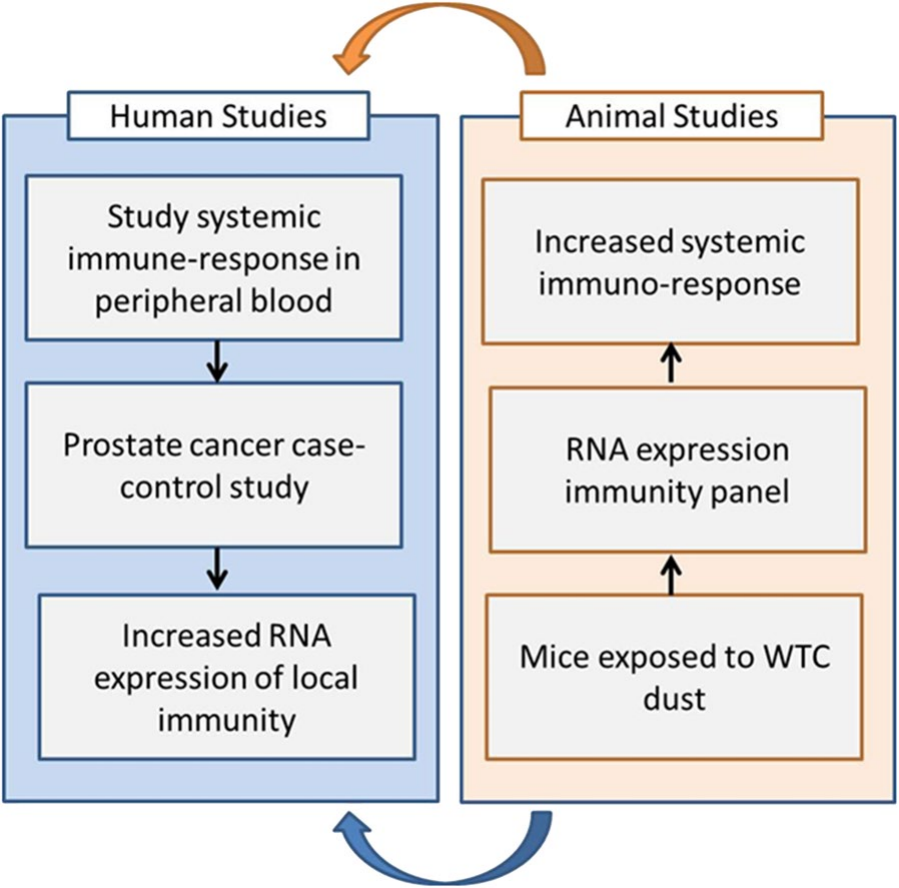
Example of studies using both human and animal samples

Following current practice, the biobank of new human and animal tissue will be used as a resource for the scientific community (Fig. 3). The human and animal tissue banks will be managed in conjunction, and will follow the same process for receiving requests of samples from qualified applicants for research purposes, and for ongoing evaluation of the bank’s utilization. Examples of questions that can be addressed include: the association between WTC exposure to specific carcinogens and unique biological markers of cancer initiation and progression; the study of genetic markers in relation to cancer characteristics and aggressiveness; biological differences between cancers in WTC responders and in their unexposed counterparts, and between cancers developed in recent years versus those developed immediately after the WTC disaster; and comparison of the systemic and local response to WTC dust in cancer-prone animal models and in WTC responders with cancer.

### Examples of studies utilizing the tissue bank resource

Of the 280 cancer cases stored in the biobank, 21 are head/neck cancer cases of the larynx, lip, nasopharynx, other oral cavity and pharynx, parotid gland, salivary gland, tongue, and tonsil.

These samples have been implemented in a pilot project to test biomarkers for DNA methylation and HPV-genotyping from WTC exposed and unexposed cases. This collaboration is between Rutgers, the State University of New Jersey, Moffitt Cancer Center, and the World Trade Center Biobank at Mount Sinai.

WTC first responders have a significant increase in risk of developing thyroid cancer. Together with Johns Hopkins, the ITE and the World Trade Center Biobank at Mount Sinai are conducting a research project to investigate if over-diagnosis of malignant thyroid cancer occurred among WTC responders by using IHC to test molecular markers of thyroid malignancy in archived thyroid cancer tissue from WTC- and non-WTC thyroid tissues.

An increased risk of prostate cancer was reported in the WTC responders and recovery workers cohort but the association with WTC-related exposures remains unknown. The ITE and the World Trade Center Biobank at Mount Sinai conducted a proof of principle study of the feasibility of inflammatory biomarkers of prostate cancer using DNA and RNA sequencing. The results of this study will be compared with a parallel study investigating tumor promotion by WTC dust in genetic and metastatic prostate cancer models conducted in collaboration with the Department of Oncological Sciences at Mount Sinai.

## Discussion

This is the first attempt to organize a bank of tissue from cancer patients diagnosed after the WTC disaster. Efforts thus far have been concentrated on building the WTCHP and other WTC surveillance programs, organizing participant follow-up, and reporting on cancer incidence through linkage with cancer registries. It is only now, more than 15 years after the attack, that cancer is becoming an important potential health consequence. Since the time frame after the exposure is becoming etiologically relevant for cancer, and the aging cohort is entering a time in life when cancer becomes more frequent, pertinent research can be conducted.

Research has estimated an increased cancer burden among WTC-exposed FDNY RRW compared to a demographically similar NYC cohort in the period January 1st 2012 to December 31st, 2013 [37]. This analysis projects an additional 2960 cancer cases (95% CI 2883–3037), with elevated estimates among white men for prostate (1437 [95% CI 1383–1495], thyroid (73 [95% CI 60–86] and melanoma (201 [95% CI 179–223] [37].

Etiologic studies demand the availability of properly prepared and stored tissues, making the tissue biobank of WTC samples a necessity for past and incident cancer cases in the future. By law, hospitals and doctors’ offices are only required to keep histopathology slides for 10 years, after which they can be disposed of [29]. Establishing a biobank of these tumor samples ensures that they will remain available for research over time, as different types of cancers become more prevalent.

This repository has valuable immediate and future use through ad hoc designed studies. The linkage with exposure data will allow studying the possible association between WTC exposure and cancer initiation and progression. The inclusion of clinical data allows for studying genetic markers in relation to cancer characteristics and aggressiveness, thus addressing the possibility that cancer in WTC responders differs from cancer in an unexposed similar population.

In order to study the effect of WTC-related exposures on clinical cancer characteristics it is necessary to conduct genomic research studies, studies of gene-environment interaction, as well as DNA methylation studies [38, 39]. The tissue bank will be able to offer biological specimens for testing; the results will be analyzed in conjunction with the epidemiologic and clinical information available from the WTCHP, as well as with the ongoing exposure study. FFPE tissue is easily utilized for translational research. DNA, RNA and proteins can all be extracted from FFPE tissue slides [40]. In fact, even after IHC, slides can be used as a DNA source [41]. This allows for a variety of research methods to be utilized, including IHC and in situ hybridization to study the morphology, DNA ploidy and high-throughput genomic assays, and RNA expression.

Finally, future studies on the biological material banked would be able to observe if cancers developed later on during the follow-up differ biologically from cancers developed in the immediate aftermath of the WTC disaster, thus helping to disentangle the role of the WTC disaster on cancer occurrence.

## Conclusions

A biobank of cancer tissue from WTC responders has been compiled with 280 specimens in storage. This tissue bank represents an important resource for the scientific community allowing for high impact studies on environmental exposures and cancer etiology, cancer outcome, and gene-environment interaction in the unique population of WTC responders.

## Abbreviations

CC: Coordinating Committee
CDC: Centers for Disease Control and Prevention
DNA: deoxyribonucleic acid
FFPE: formalin-fixed paraffin-embedded
FDNY: Fire Department of the City of New York
H&E: hematoxylin and eosin
IHC: immunohistochemistry
IRB: Institutional Review Board
ITE: Institute for Translational Epidemiology
NIOSH: National Institute for Occupational Safety and Health
NYC: New York City
NYS: New York State
REP: research evaluation panel
RNA: ribonucleic acid
RRW: rescue and recovery workers
SIR: standardized incidence ratio
TMA: tissue microarrays
UC: Utilization Committee
WTC: World Trade Center
WTCHP: World Trade Center Health Program
